# Recent spatial aggregation tendency of rainfall extremes over India

**DOI:** 10.1038/s41598-019-46719-2

**Published:** 2019-07-16

**Authors:** Akshaya C. Nikumbh, Arindam Chakraborty, G. S. Bhat

**Affiliations:** 1 Centre for Atmospheric and Oceanic Sciences, Indian Institute of Science, Bangalore, 560012 India; 2 Divecha Centre for Climate Change, Indian Institute of Science, Bangalore, 560012 India

**Keywords:** Attribution, Atmospheric dynamics

## Abstract

Significant increase in the frequency of occurrences of rainfall extremes has been reported over several parts of the world. These extreme events were defined at individual grids without considering their spatial extent. Here, using ground-based observations over India during boreal summer, we show that the average size of spatially collocated rainfall extremes has been significantly increasing since 1980. However, the frequency of occurrences of such collocated extreme events remains unchanged. Around 90% of the total number of large-sized events (area ≥ 70 × 10^3^ km^2^) of our study period (1951 to 2015) have occurred after 1980. Some of the major floods in recent decades over India are attributed to these large events. These events have distinctive precursory planetary-scale conditions, unlike their smaller counterparts. As the underlying physical mechanisms of extremes rainfall events are size-dependent, their changing spatial extent needs to be considered to understand the observed trends correctly and obtain realistic future projections.

## Introduction

The increasing number of precipitation extremes over the past half-century are shown to be associated with the rising temperatures^1–6^. As climate changes, their spatio-temporal characteristics are likely to be modified^7^. While thermodynamic factors provide the necessary conditions for extreme rainfall^8,9^, dynamics can play a crucial role in altering such events through feedback processes^7,10,11^. Thus, the changes in temperature and circulation together may modify the spatio-temporal characteristics of rainfall extremes. The simulations of future scenarios by climate models show a decrease in the duration of mid-latitude precipitation extremes and changes in the zonal extent of both signs depending on latitude^12^. Observations confirm the changing temporal distribution of heavy rain events^13–15^. The frequency and duration of rainstorms are increasing over the Indian landmass^16^. The changes in their spatial extent, however, have not been reported so far. Here we use observational data to show that the spatial extent of rainfall extremes is increasing in a tropical region.

The very definition of rainfall extreme affects the conclusions drawn^17^ and hence, there is a need to revisit it. In past studies, when rainfall exceeded a pre-defined threshold, it was termed as an extreme event and its total count for a given region over a season/year became the number of extremes^4,18,19^. For gridded rainfall data, the total count is the number of grids having extreme rainfall. A synoptic system may produce extreme rainfall at several grids on a given day (Supplementary Fig. 1). Physically it is a single event; but traditionally, as many events would be counted as the number of grids having extreme rainfall. Thus, the number of grid counts should not be equated with the number of events when there is a sense of co-location in space and time. The spatial coherence of an extreme event needs to be taken into consideration in defining an extreme event.

## Results

We identify the spatially-adjacent grids that are simultaneously experiencing rainfall crossing the threshold, and treat it as a single extreme rainfall event (ERE; details in Methods). The total number of grids having extreme rainfall (*N*_T_) and the number of EREs (*N*_E_) in a season are related by ${N}_{{\rm{T}}}={\sum }_{k=1}^{{N}_{{\rm{E}}}}{S}_{{\rm{k}}}$, where *S*_k_ is the size of the *k*^th^ ERE measured in grid units. It is to be noted that past studies based on the gridded rainfall data^4,18,19^ reported *N*_T_. Neglecting the spatial connectivity of extreme events leads to an overestimation of their number and an incorrect trend. For example, the observed trend in the number of rainfall extremes over Central India based on *N*_T_ is 0.34 yr^−1^ while that in *N*_E_ is 0.13 yr^−1^ (Supplementary Fig. 2a). Several studies have reported a significant rise in the frequency and magnitude of extreme rainfall events over Central India^4,18,19^. We focus on this area to facilitate a comparison with the past results.

**Figure 1:**
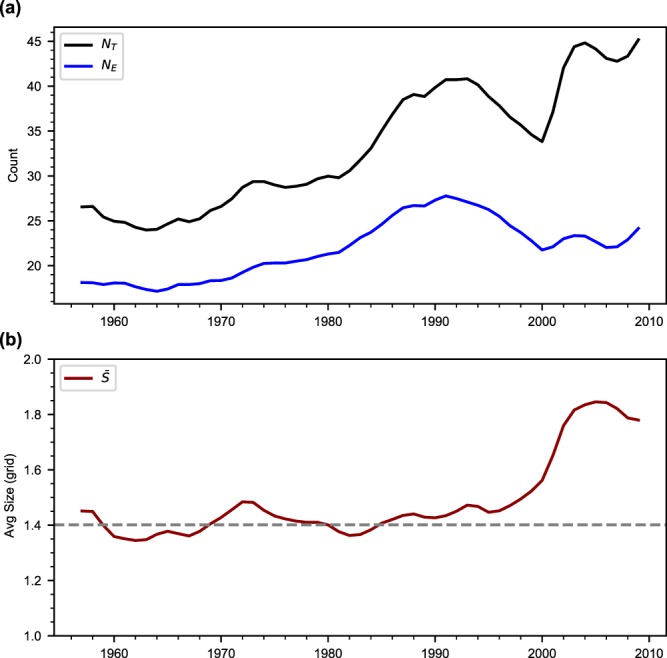
Temporal variation of extreme rainfall events over Central India. Smoothed time series of: (**a**) total number of 1° × 1° grids with extreme rainfall in a summer monsoon season (*N*_*T*_) and the number of extreme rainfall events (*N*_*E*_); (**b**) average size ($\bar{S}$) of extreme rainfall events. The dashed line is 1951–1980 period average. Time series shown are 11-year moving averages smoothed using a 3 point (0.25, 0.5 and 0.25) weighted averaging to highlight the long-term variations. Time series (without smoothing) of *N*_*T*_ and $\bar{S}$ show a significant increasing trend at the 99% confidence level based on the Mann-Kendall test. The *N*_T_ is equivalent to the count of extreme events reported in the past studies^4,6,18^. The analysis used the gridded rain gauge data of the India Meteorological Department^31^.

The changing contribution of *N*_E_ to *N*_T_ is evident in recent decades (Fig. 1a). The *N*_T_ shows a large increase between 1980 and 1990 followed by a decrease and an increase, respectively, in the next two decades. It suggests interdecadal variations of *N*_T_ superimposed on its overall increasing trend. The *N*_E_ follows the *N*_T_ trend up to 1990 and the difference between *N*_E_ and *N*_T_ increases thereafter. The average size ($\bar{S}$) of EREs for a season is given by $\bar{S}=\frac{{N}_{{\rm{T}}}}{{N}_{{\rm{E}}}}$. The growing difference between *N*_T_ and *N*_E_ after 1990 is due to the increasing contribution by $\bar{S}$ to *N*_T_ (Fig. 1b). The $\bar{S}$ shows a significant rise after 1980 (Supplementary Fig. 2b). One important point to note here is that since 1990, the number of extreme events has come down but their average size ($\bar{S}$) has increased, resulting in the rise of *N*_*T*_. To further quantify the changing contribution of *N*_E_ and $\bar{S}$ to *N*_T_, we look at the fractional changes that can be written as,

1 $$\frac{d{N}_{{\rm{T}}}}{{N}_{{\rm{T}}}}=\frac{d\bar{S}}{\bar{S}}+\frac{d{N}_{{\rm{E}}}}{{N}_{{\rm{E}}}}$$

During the study period, the contribution to the fractional changes in *N*_T_ by the fractional changes in $\bar{S}$ is 31%. The remaining 67% and 2% changes are due to the fractional changes in *N*_E_ and a nonlinear term respectively (See methods for details). In the last two decades, the rise in *N*_T_ is mainly due to the increasing average size (Supplementary Fig. 3).

**Figure 2:**
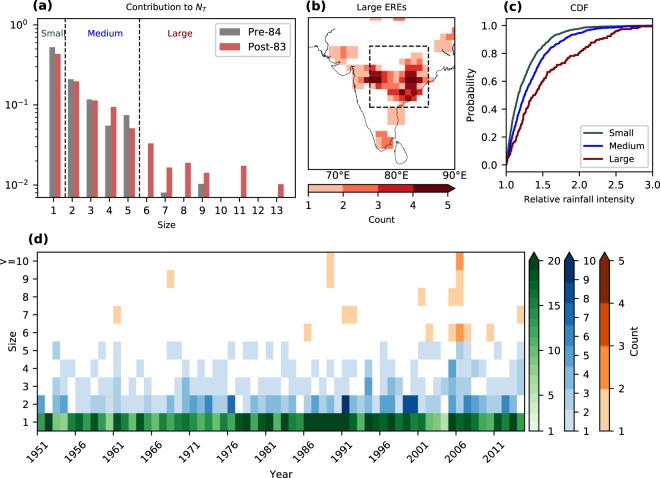
Characteristics of extreme rainfall events. (**a**) Contribution of the events of different sizes to the total number of grids having extreme rainfall events (*N*_*T*_) during the pre-84 (1952–1983) and post-83 (1984–2015) period. Based on the size, different types of events are defined as small (size 1), medium (size 2–5) and large (size ≥ 6) Extreme Rainfall Events (EREs). (**b**) Spatial distribution of large EREs. (**c**) Cumulative distribution function (CDF) of relative rainfall intensity of EREs. (**d**) Year-wise count of the extreme rainfall events of various sizes. The CDFs are significantly different from each other at the 99% confidence level (using the Kolmogorov–Smirnov test). The inset box in Fig. 2b indicates the study region (Central India: 15°–25°N, 75°–85°E).

To understand the changes in the distribution of EREs, we divide the study period into two equal intervals: 1952–1983 (pre–84) and 1984–2015 (post–83). The contribution to *N*_T_ by bigger-sized EREs has increased post–83 (Fig. 2a). In fact, there were only two events of size greater than 5 in the pre–84 period and their contribution to *N*_T_ does not stand out. The likelihood of getting a bigger size ERE has increased post–1983 (Supplementary Fig. 4).

Over India as a whole, small, medium and large EREs show disparate geographical preferences. The area that falls in the path of monsoon systems, i.e., monsoon trough^20,21^ is more likely to experience large and medium EREs (Fig. 2b, Supplementary Fig. 5a). The large EREs are more frequent in the southern sector of the monsoon trough (Fig. 2b). The windward side of the Western Ghats is a favourable area for small EREs to occur (Supplementary Fig. 5b). This follows the observation that small-sized convective systems dominate the Western Ghats region^22^.

The large events are more intense as well. The relative rainfall intensity is defined as the ratio of an actual rainfall to the threshold value. The average relative rainfall intensities of large, medium and small EREs are 1.5, 1.3 and 1.2 respectively. The large events mainly consist of grids with higher relative rainfall intensity than the small and medium events (Fig. 2c). The fraction of grids crossing the relative rainfall intensity of 1.5 are 15%, 24% and 40% respectively for small, medium and large EREs.

Out of the 20 large EREs that occurred during the study period, 90% of them are post–1980 and 65% are post–2000 (Fig. 2d). The medium events show a rise in number mainly after 1990. The small events show a peak between the mid-1970s and the early 1990s. Over the study period, medium and large EREs show a significant rising trend (Supplementary Fig. 6). However, small EREs do not show any significant trend.

**Figure 3:**
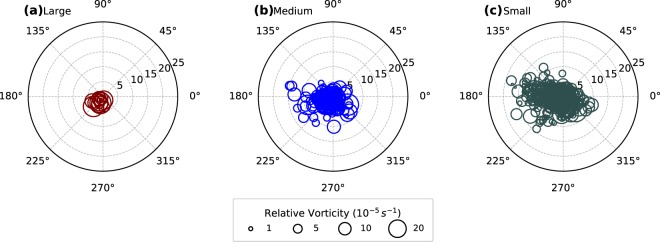
Spatial distribution of rainfall extremes with respect to monsoon low pressure systems (LPSs). Relative locations of the centre of mass of (**a**) large, (**b**) medium and (**c**) small Extreme Rainfall Events (EREs) with respect to the LPS centre (taken as position of the 850 hPa relative vorticity maxima). The origin of the polar plot and bubbles represent the LPS centre and the centre of mass of EREs respectively. The radial distance from the origin is in degrees. The area of the bubble is directly proportional to the relative vorticity (850 hPa) at the LPS centre. For this analysis, the LPSs over the Bay of Bengal and Central India (10°–27°N, 75°–95°E) are considered for the period 1979–2012. The archived LPS track data by Hurley and Boos^23^ is used for this analysis.

Are there synoptic and global circulation features that are more conducive for EREs? To understand the association of EREs with synoptic conditions, we examined if a low pressure system (LPS) was present in the region when an ERE occurred by taking the track data of Indian monsoon LPS^23^. It is found that LPSs were present on 83%, 92% and 100% of the time when small, medium and large EREs occurred. The large EREs occur close to the centre of the LPS (within 400 km) with preference to the South-western sector (Fig. 3a), climatologically known to experience maximum precipitation in LPS^20,21^. The small and medium EREs have no preferred direction within the first 500 km from the centre of the LPS (Fig. 3b,c). Beyond 500 km, they are clustered more on the western side of the LPS. The large EREs occurred only when the 850 hPa vorticity at the centre of the LPS exceeded 2.8 × 10^−5^ s^−1^, unlike small and medium events.

**Figure 4:**
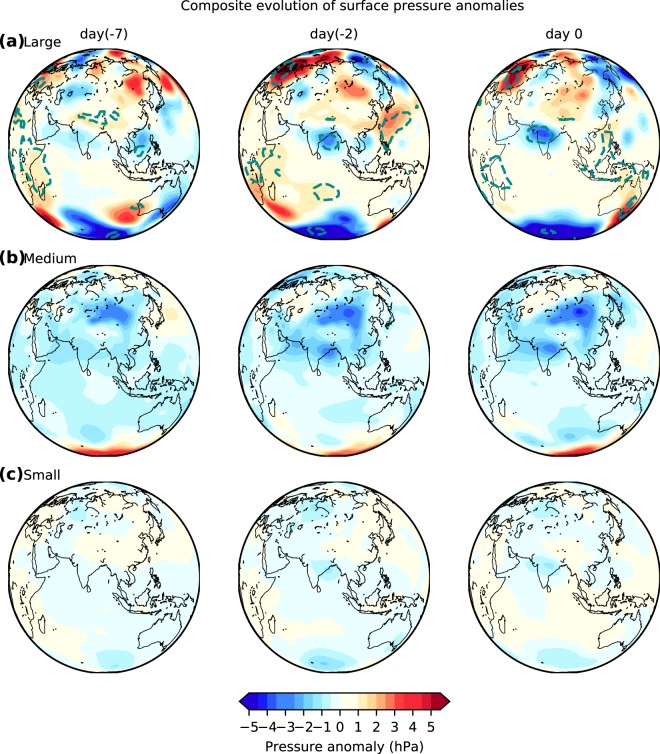
Surface pressure anomalies associated with rainfall extremes. Composite evolution of daily surface pressure anomalies for (**a**) large (**b**) medium and (**c**) small extreme rainfall events. Here, day 0 is the day of the Extreme Rainfall Event; day(−2) and day(−7) respectively indicate two and seven days before the event. The dotted contours indicate the regions that have significant anomalies at the 99% confidence level (using t-test). The data is obtained from the National Centers for Environmental Prediction (NCEP) reanalysis for the period 1951–2015.

We take the surface pressure anomaly as a proxy for planetary-scale circulation and consider the anomalies 7 days prior (day(−7)) to the day of the ERE (day 0). When large EREs occur, strong positive and negative pressure anomaly patterns are seen in the middle latitudes even a week before that gradually intensifies until the event day (Fig. 4a). The small and medium EREs show negative anomalies over the entire Indian subcontinent and adjacent regions (Fig. 4b,c). The negative anomalies over the Bay of Bengal and the Indian subcontinent suggest the development of an LPS over the Bay and then, its westward movement onto the subcontinent (Fig. 4a–c). The surface pressure anomalies indicate that the LPSs leading to the large EREs are assisted by the planetary scale conditions, which is not observed for the small and medium EREs.

## Discussion

Our study highlights the importance of the definition of rainfall extremes. We show that a lack of consideration of the spatial extent of rainfall extremes overestimates their count and trend. A spatial organization of rainstorms affects the thermodynamic structure of the atmosphere. For example, clustered thunderstorms lead to more drying of the atmosphere and warming in the lower troposphere than randomly-distributed thunderstorms^24^. We speculate that increasing size of precipitation extremes would impact the thermal structure of the atmosphere. Hence, the spatio-temporal distribution of precipitation extremes should also be considered in addition to their magnitude and frequency.

The large and intense events are of great concern as they are likely to cause widespread flooding. As an example, one of the largest events during the study period occurred on 24 July 1989. This event had devastating effects on life and property; approximately 2500 people were killed and train services were disrupted for more than three weeks in many places^25^. The large events are mainly associated with LPSs and have distinctive precursory planetary-scale conditions. It has been observed that monsoon lows and severe cyclonic storms show an increasing trend^26,27^. An improved understanding of the formation and propagation of synoptic systems during the monsoon season would help in taking control measures. The observed changes in the distribution of heavy rainfall events should be considered to plan infrastructure and resource management for a robust adaptation to future changes in precipitation extremes.

This analysis opens up many interesting questions. We find that large events over Central India are mainly observed post–1980. This is coincident with the observed weakening of large-scale monsoon circulation and rapid warming of the equatorial Indian Ocean^28–30^. It is intriguing to investigate the impact of these changes on the size and distribution of rainfall extremes, and finding feedback mechanisms that trigger and sustain intense and larger convective systems. A possible interlink between the obtained results and organized convection could prove very useful in reducing uncertainty in future projections of precipitation extremes.

## Methods

### Classification of rainfall extremes

The daily gridded rainfall data of the India Meteorological Department (IMD) rain gauge network at 1° × 1° spatial resolution^31^ for the period 1951–2015 is used. The dataset is developed from 2,140 rain-gauge stations. It uses the Shepard interpolation technique considering the directional effects and barriers. Multi-stage quality control of dataset is carried out before the interpolation. This dataset uses a fixed rainfall network so that it can be used for examining long term rainfall trends^18^.

The study period is the boreal summer monsoon season, i.e., June to September. An extreme rainfall event occurs at a grid when the daily rainfall exceeds a threshold. Within India, the monsoon seasonal rainfall varies from less than 100 mm to more than 3,000 mm (Supplementary Fig. 7a). Therefore, using a single threshold for all the grids is not ideal. For each grid, it is taken as the 99.5^th^ percentile value calculated considering the entire data period and all rainy days with intensity exceeding 1 mm/day between Julian days 151 and 273 (1 June to 30 September in non-leap years). Some rain shadow areas of the Western Ghats and Northwest India receive very little rain during June–September; the number of rainy days are small and the 99.5^th^ percentile is less than 30 mm day^−1^. Comparatively, on the windward side of the Western Ghats, the threshold is more than 150 mm day^−1^ (Supplementary Fig. 7b). The severity of extreme rainfall events at such vastly-different thresholds is not comparable; the 30 mm day^−1^ rainfall is unlikely to cause damage to life or property compared to the 150 mm day^−1^ rainfall. In view of this, the threshold is taken as larger of the 99.5^th^ percentile and 50 mm day^−1^. In the area selected for detailed analysis, i.e., Central India, the threshold is greater than 50 mm day^−1^ and the results are not affected by this choice.

The grids having a common boundary, where extreme events occur simultaneously are identified using the connected component labelling algorithm^32^. This algorithm gives labelled objects as output. All the connected grids share the same label and are treated as a single event. Thus, each labelled object corresponds to an extreme rainfall event (ERE) whose size is the number of grids that it occupies. These events are classified into small (size 1), medium (size 2–5) and large (size ≥ 6). Central India (15°–25°N, 75°–85°E) is the main study area (boundaries shown in Fig. 2b). The EREs for entire India are first obtained; an ERE belongs to Central India if at least one grid of the event lies within the Central Indian domain. This is done to account for situations where an ERE extends outside the boundaries of Central India. The choice of different fixed thresholds such as 120 mm day^−1^ or 100 mm day^−1^ give similar results (Supplementary Figs. 8 and 9).

The year 2006 was an unusual year where a large number of long-lived low pressure systems had formed, resulting in the highest number of large EREs observed during the study period. The mean number of LPSs over the study region during 1979 to 2012 is 15 (using Hurley and Boos dataset). The number of LPSs in the year 2006 was 22. The large-scale conditions and internal feedbacks that possibly favor long-lived low-pressure systems are investigated in the earlier studies^33^. The year 2006 was a positive IOD year. The positive IOD events provide a conducive environment for increased low-pressure systems activity by strengthening the cross-equatorial moisture transport from south-eastern tropical Indian Ocean into the Bay of Bengal and by enhancing the barotropic instability of monsoon flow. In order to cross-verify trends, we have checked the trends of $\bar{S}$ and *N*_*T*_ excluding the year 2006 and results show significant trends even without the year 2006.

The results are also cross verified with the GPCP 1DD v1.2 dataset (Supplementary Fig. 10). It is a daily dataset with 1° × 1° degree spatial resolution. Similar to the IMD dataset, the 99.5^*th*^ percentile threshold is used to identify the extreme events using the GPCP dataset. Time series of *N*_*T*_ is analyzed over Central India for the common period of 1998 to 2014. The GPCP dataset shows similar interannual variability of EREs as observed in the IMD dataset, including the peak of 2006. The GPCP dataset uses geostationary infrared satellite imagery along with direct gauge measurements over land to determine daily precipitation rates. The merged datasets such as GPCP have a tendency to introduce a spatial and temporal averaging effect upon the data^34^. This possibly explains the lower amplitude of interannual variation for the GPCP dataset compared to the IMD dataset.

### Contribution of size and count to the trend of rainfall extremes

The equation (1) in the discrete domain can be represented as:

2 $$\begin{array}{rcl}\frac{{N}_{{\rm{T}}({\rm{m}}+1)}-{N}_{{\rm{T}}({\rm{m}})}}{0.5\times ({N}_{{\rm{T}}({\rm{m}}+1)}+{N}_{{\rm{T}}({\rm{m}})})} & = & \frac{{\bar{S}}_{({\rm{m}}+1)}-{\bar{S}}_{({\rm{m}})}}{0.5\times ({\bar{S}}_{({\rm{m}}+1)}+{\bar{S}}_{({\rm{m}})})}\\  &  & +\,\frac{{N}_{{\rm{E}}({\rm{m}}+1)}-{N}_{{\rm{E}}({\rm{m}})}}{0.5\times ({N}_{{\rm{E}}({\rm{m}}+1)}+{N}_{{\rm{E}}({\rm{m}})})}\\  &  & +\,O({\rm{\Delta }}\bar{S},{\rm{\Delta }}{N}_{{\rm{E}}})\end{array}$$

where *m* is a set of years and $O({\rm{\Delta }}\bar{S},{\rm{\Delta }}{N}_{E})$ is a nonlinear residual term. To calculate the fractional change in *N*_T_ for the period 1952–2015, we consider two equal intervals, 1952–1983 (*m*) and 1984–2015 (*m* + 1). Similarly, the decadal fractional changes are calculated considering the sets that divide the entire study period into equal intervals (Supplementary Fig. 3).

### Significance test

The non-parametric Mann-Kendall test is used for trend analysis. It is suitable for rainfall data as it does not assume the underlying distribution. To detect the significance of anomalies, we used the two-sided t-test. A comparison of the cumulative distribution functions (CDFs) is done using the Kolmogorov–Smirnov test (K–S test). The two sample K–S test is separately conducted for small and large, medium and large, and small and medium EREs. The results for all pairs suggest that the CDFs are significantly different from each other at 99% confidence level.

### Association with LPS

In order to examine the association between EREs and low pressure systems (LPS), the track data of LPSs by Hurley and Boos^23^ that is available for the period 1979–2012 is taken. Hurley and Boos identify LPSs using the ERA-Interim 850 hPa relative vorticity, mean sea-level pressure and surface (10 m) wind speed, and includes monsoon lows, depressions, deep depressions and above. Our analysis is based on the presence of the nearest LPS (within domain: 10°–27°N, 75°–95°E) when an extreme rainfall event occurred.

The results are cross-verified with the track data of the Monsoon depressions that includes monsoon depression (MD), Cyclonic Storms and Severe Cyclonic storms. This dataset doesn’t include monsoon lows (systems with surface wind speed < 17 Knots). The distribution of EREs with respect to the monsoon depression is obtained (Supplementry Fig. 11) using this dataset for the period 1979 to 2012. Overall preference of the southwestern sector for the large events and western-side preference for the small events is similar. Out of 17 large EREs, 15 events were associated with the MDs instead of all events (as observed in case of the LPS track data by Hurley and Boos). It is found that MDs were present on 27% (43%) the time when small (medium) EREs occurred. We understand this difference in statistics between MD and LPS is possible as the IMD monsoon depression dataset doesn’t include the monsoon lows, while the LPS data by Hurly and Boos includes all viz., monsoon lows, depressions, deep depressions and above.

## Supplementary information

Supplementary Information

## Data Availability

The rainfall data used in the study is obtained from the IMD (http://www.imd.gov). The global monsoon low pressure systems track dataset is available at http://worldmonsoons.org/global-monsoon-disturbance-track-dataset/. The mean sea level pressure dataset can be downloaded from NOAA NCEP website (https://www.esrl.noaa.gov/psd/data/gridded/data.ncep. reanalysis.html).

## Code Availability

The Python codes used for the analysis in this study are available on request from the author A.C.N., nikumbh@iisc.ac.in.
