# Supplementary material for: Recent spatial aggregation tendency of rainfall extremes over India
Source: Sci Rep. 2019 Jul 16;9:10321. doi: 10.1038/s41598-019-46719-2 (PMC6635486; doi:10.1038/s41598-019-46719-2)
Supplement: Supplementary file 1 — Supplementary Information [file 41598_2019_46719_MOESM1_ESM.pdf]

Supplementary Information for ”Recent spatial aggregation tendency of rainfall extremes over India”

Akshaya C. Nikumbh[1,2], Arindam Chakraborty[1,2*] & G. S. Bhat[1,2]

[1]*Centre for Atmospheric and Oceanic Sciences, Indian Institute of Science, Bangalore, 560012, India.*

[2]*Divecha Center for Climate Change, Indian Institute of Science, Bangalore, 560012, India.*

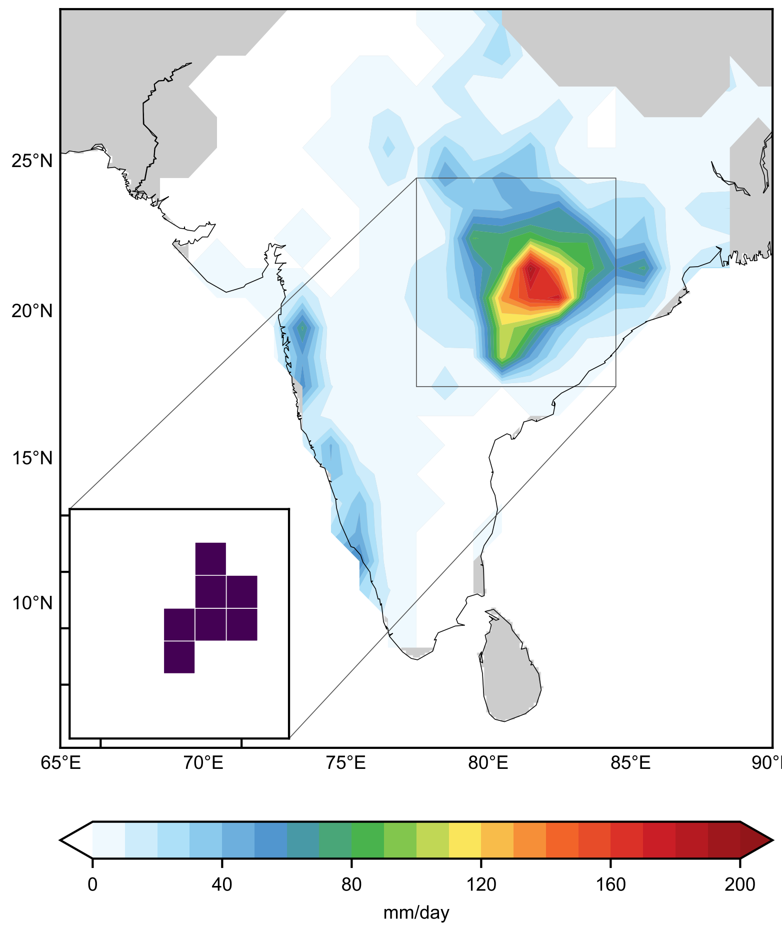

Supplementary Fig. 1. An example of the extreme rainfall event that occurred on 22 July 2014, covers seven $1^\circ \times 1^\circ$ grids. In this study, it is identified as a single event of the size 7 (shown in the inset box) rather than 7 different events by the method used in the past studies. This event contributed 7 and 1 to N_T and N_E, respectively.

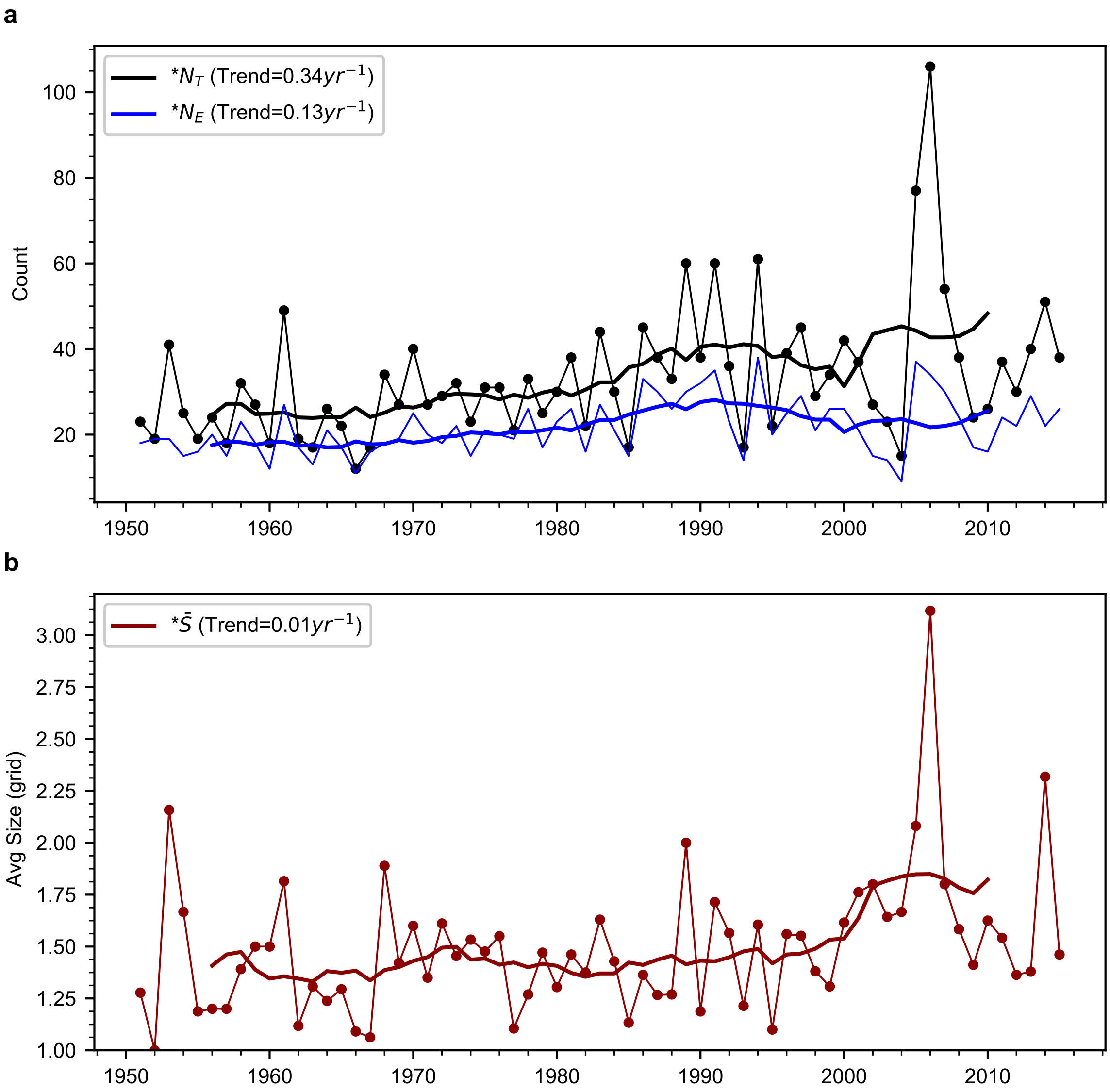

Supplementary Fig. 2. Time series of **a.** the total number of $1^\circ \times 1^\circ$ grids with extreme rainfall (N_T), the number of extreme rainfall events (N_E), and their **b.** average size ($\bar{S}$) over Central India. The smoothened curves on the time series plots represent 11-year moving averages. Asterisks(*) indicate the increasing trends are significant at the 99% confidence level using the Mann-Kendall test.

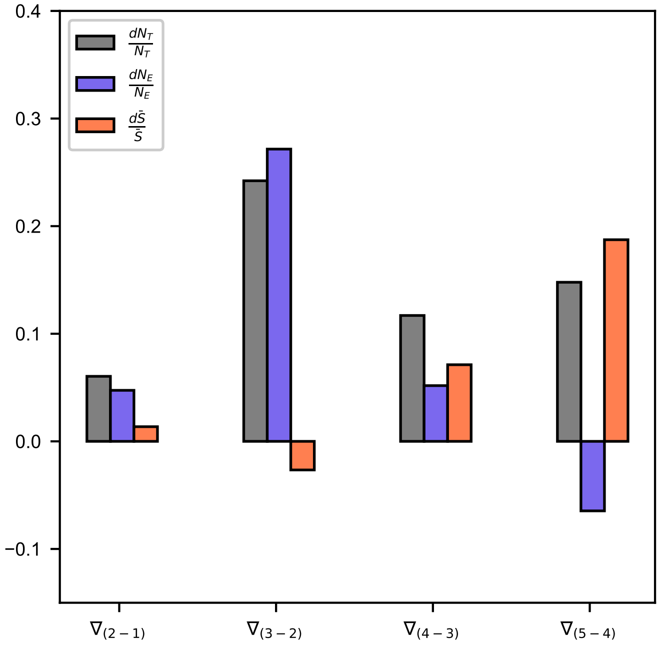

Supplementary Fig. 3. The contribution by fractional changes in the average size ($\frac{d\bar{S}}{\bar{S}}$) and the count ($\frac{dN_{\mathrm{E}}}{N_{\mathrm{E}}}$) to the total number of $1^{\circ} \times 1^{\circ}$ grids with extreme rainfall ($\frac{dN_{\mathrm{T}}}{N_{\mathrm{T}}}$) for different sets (m) of years (Sets: 1951-1964,1964-1977, 1977-1990,1990-2003, 2003-2015). $\nabla_{(\mathrm{m+1-\,m})}$ indicates fractional changes calculated from consecutive sets $(m + 1, m)$ (See method section for details).

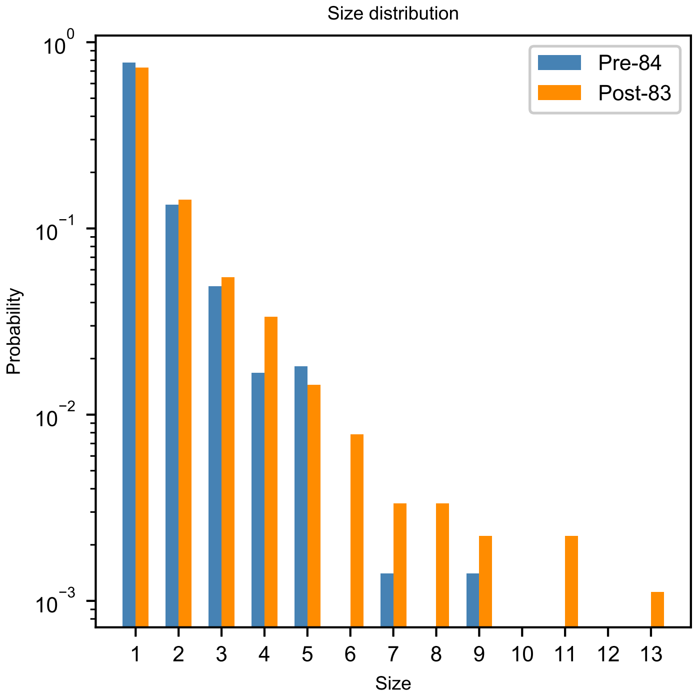

Supplementary Fig. 4. Normalized size distribution of extreme rainfall events during the pre-84 (1952-1983) and post-83 (1984-2015) periods.

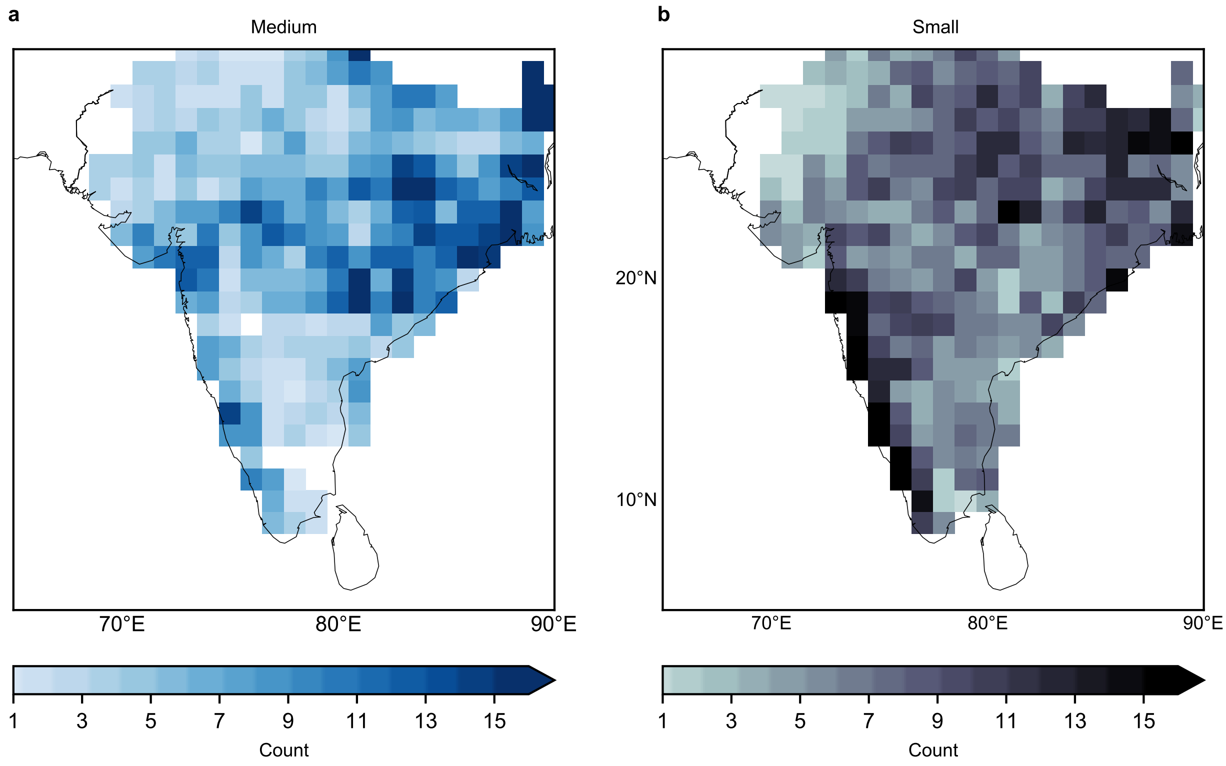

Supplementary Fig. 5. Spatial distribution of **a.** medium and **b.** small extreme rainfall events

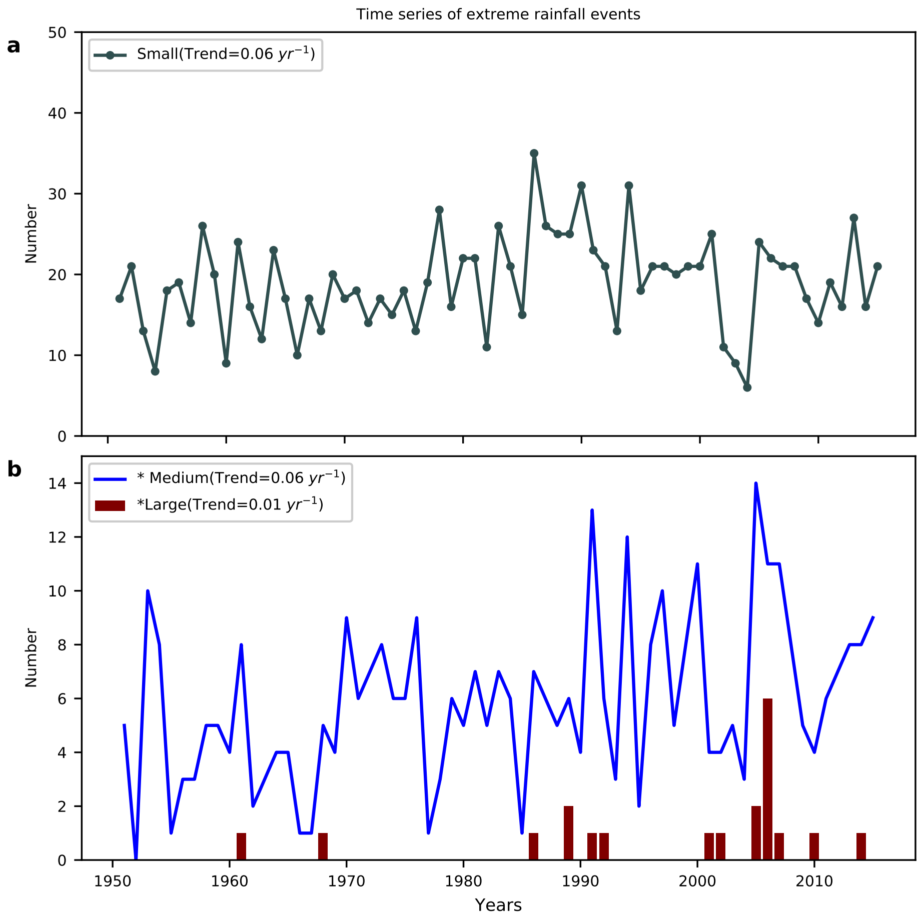

Supplementary Fig. 6. Time series of small, medium and large extreme rainfall events. Asterisks(*) indicate the increasing trends are significant at the 95% confidence level using the Mann-Kendall test.

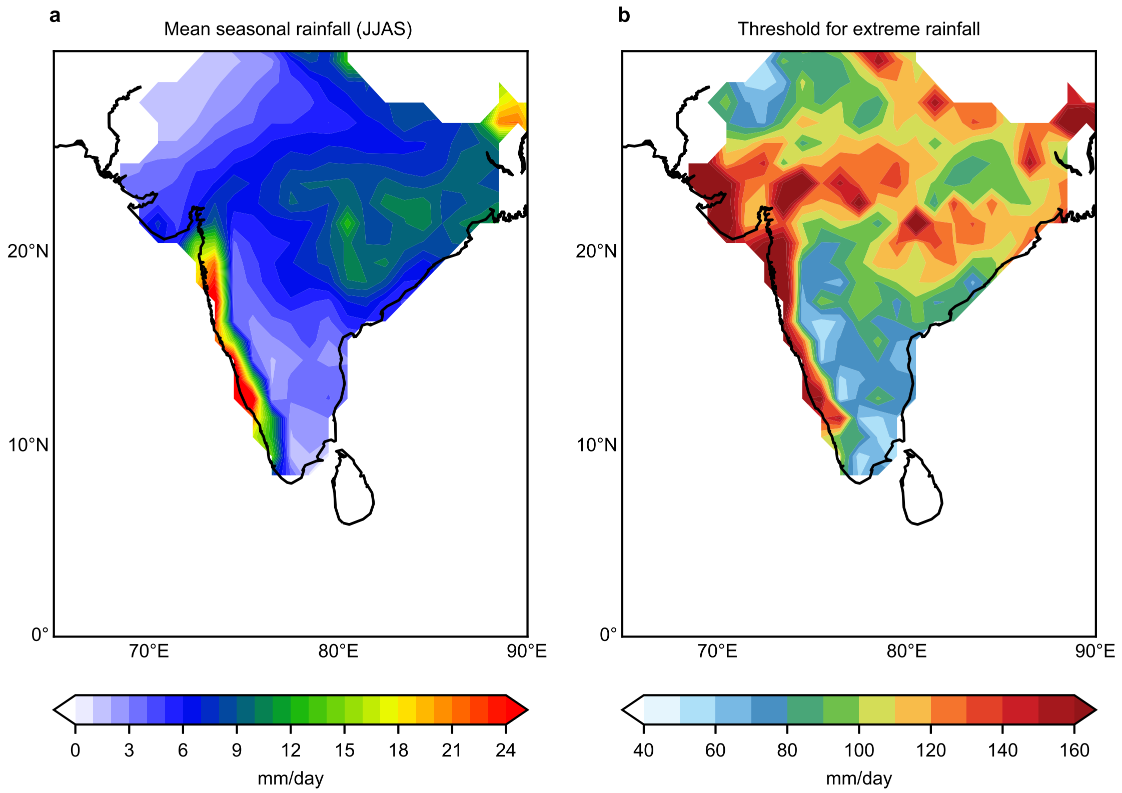

Supplementary Fig. 7. a. Climatology of daily rainfall during the Indian summer monsoon **b.** 99.5th percentile threshold. Both are obtained using JJAS rainfall data for the period 1951-2015.

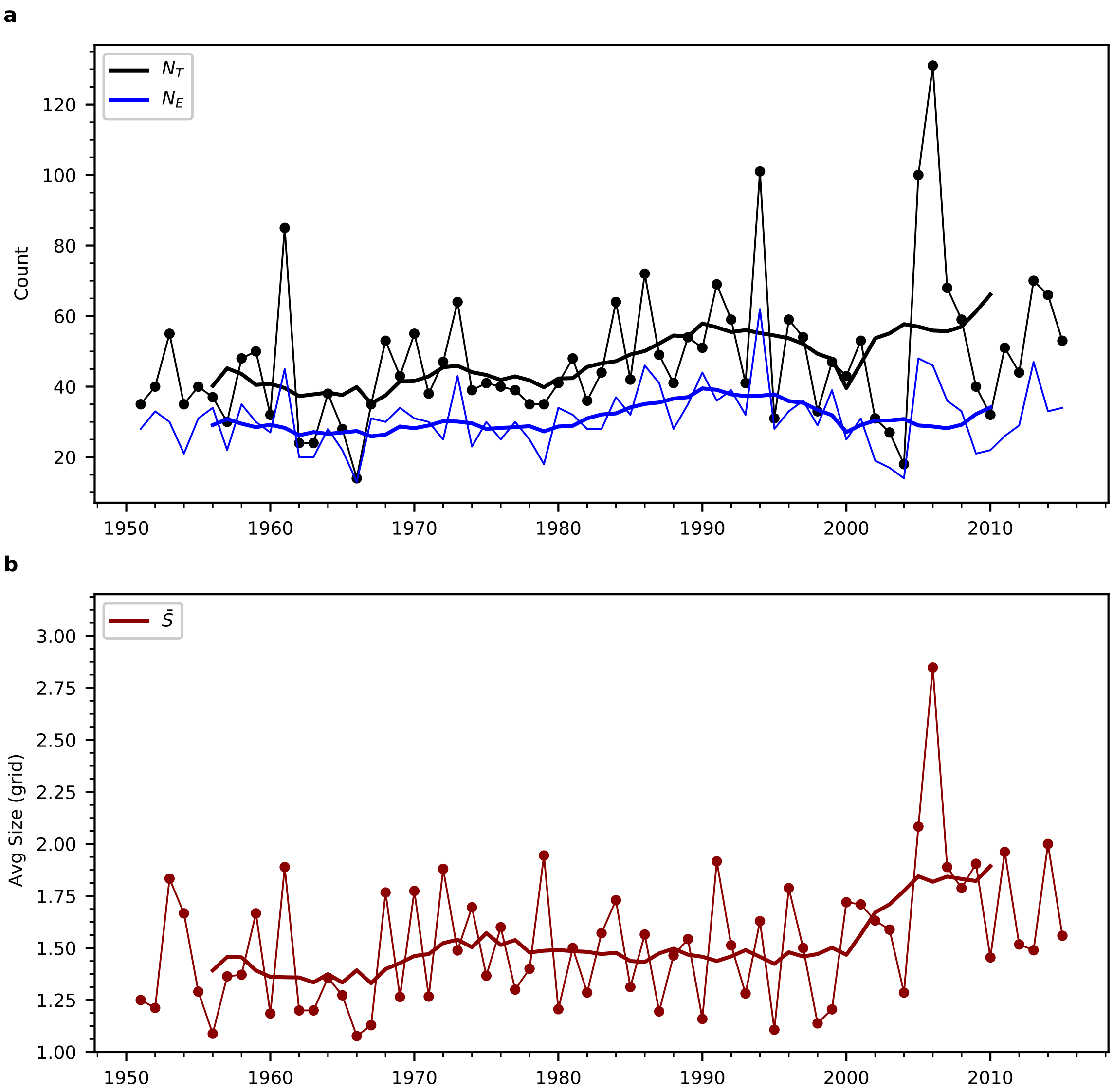

Supplementary Fig. 8. Same as Supplementary Fig. 2. but for a fixed threshod of 100 mm day^{-1}.

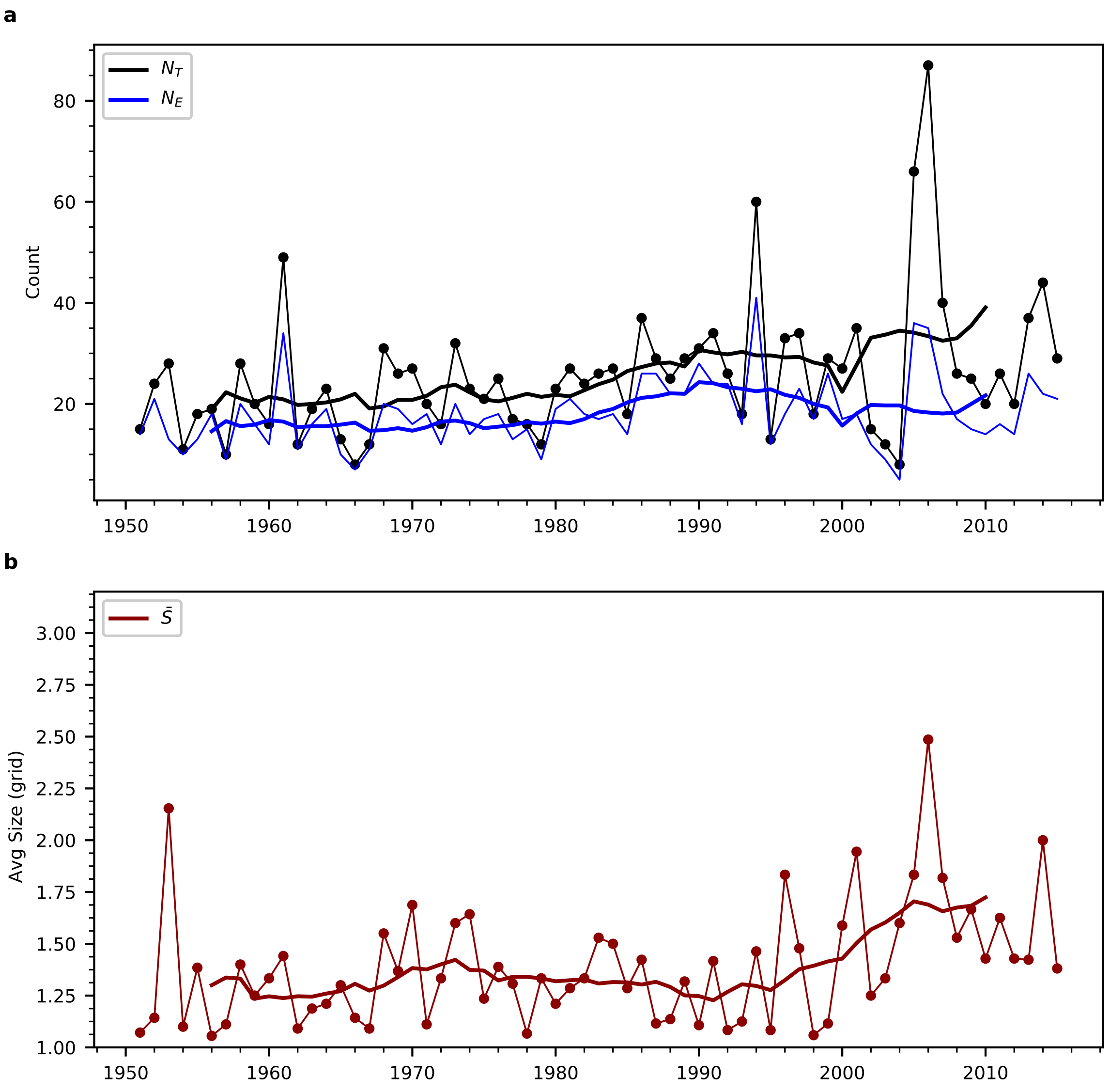

Supplementary Fig. 9. Same as Supplementary Fig. 2. but for a fixed threshod of 120 mm day^{-1}.

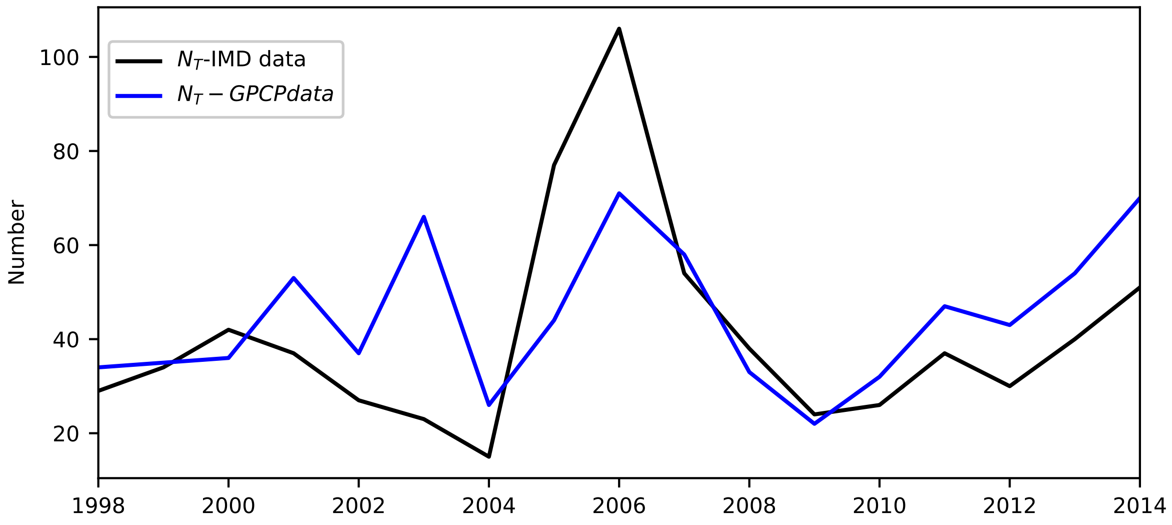

Supplementary Fig. 10. Time series of N_T using the GPCP 1DD v1.2 dataset and the IMD dataset.

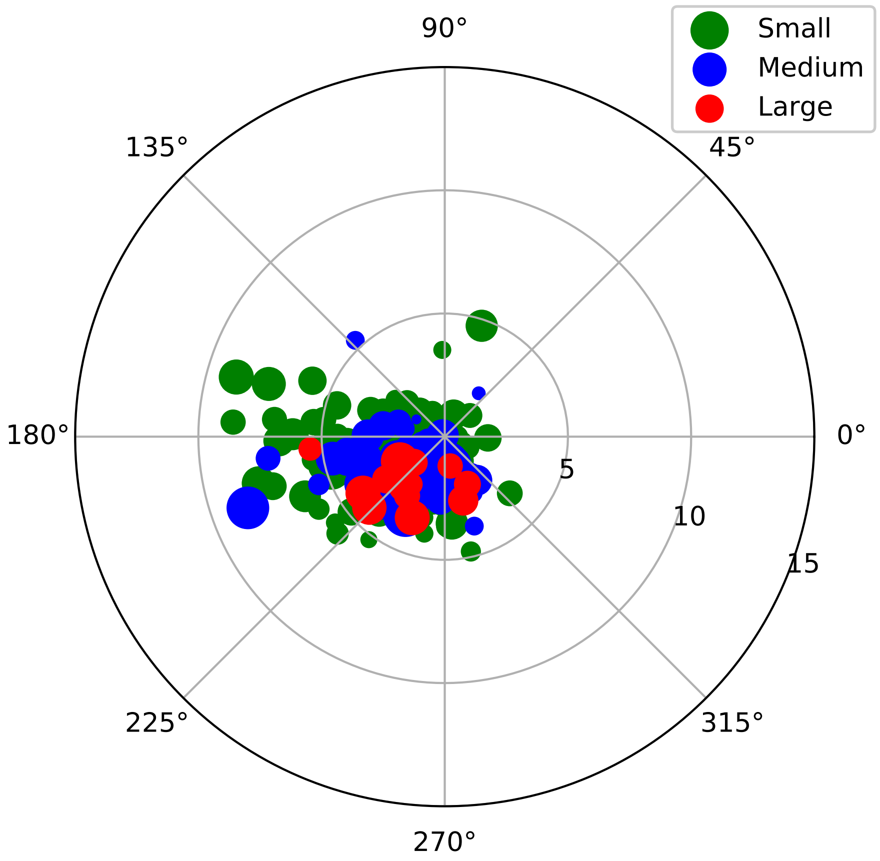

Supplementary Fig. 11. The relative locations of Small, medium and large EREs relative to the Monsoon depression.
